# Socioeconomic representativeness of Australian, Canadian and British cohorts from the paediatric diabetes AdDIT study: comparisons to regional and national data

**DOI:** 10.1186/s12916-023-03222-w

**Published:** 2023-12-20

**Authors:** Farid H. Mahmud, Antoine B. M. Clarke, Yesmino Elia, Jacqueline Curtis, Paul Benitez-Aguirre, Fergus J. Cameron, Scott T. Chiesa, Cheril Clarson, Jennifer J. Couper, Maria E. Craig, R. Neil Dalton, Denis Daneman, Elizabeth A. Davis, John E. Deanfield, Kim C. Donaghue, Timothy W. Jones, Sally M. Marshall, Andrew Neil, M. Loredana Marcovecchio

**Affiliations:** 1 Division of Endocrinology, Department of Paediatrics, The Hospital for Sick Children and University of Toronto, 555 University Avenue, RM 5446 Black Wing, Toronto, ON M5G 1X8 Canada; 2 Institute of Endocrinology and Diabetes, The Children’s Hospital at Westmead, University of Sydney, Sydney, Australia; 3 Department of Paediatrics, University of Melbourne, Melbourne, Australia; 4 Institute of Cardiovascular Science, University College London, London, UK; 5 London Health Sciences Center, London, ON Canada; 6 Departments of Endocrinology and Diabetes and Medical Imaging, Women’s and Children’s Hospital, Adelaide, Australia; 7 Discipline of Paediatrics & Child Health, School of Clinical Medicine, University of New South Wales Medicine & Health, Sydney, Australia; 8 Evelina London Children’s Hospital, Guy’s and St Thomas’ NHS Foundation Trust, London, UK; 9 Telethon Kids Institute, University of Western Australia, Perth, Australia; 10 Faculty of Clinical Medical Sciences, Diabetes Research Group, Translational and Clinical Research Institute, Newcastle University, 4Th Floor William Leech Building, Framlington Place, Newcastle Upon Tyne, UK; 11 Oxford Centre for Diabetes, Endocrinology & Metabolism, University of Oxford, Oxford, UK; 12 Department of Paediatrics, University of Cambridge, Cambridge, UK

**Keywords:** Paediatric, Type 1 diabetes, Multicenter trials, Clinical trial, Socioeconomic status, Deprivation, Marginalization

## Abstract

**Background:**

Given limited data regarding the involvement of disadvantaged groups in paediatric diabetes clinical trials, this study aimed to evaluate the socioeconomic representativeness of participants recruited into a multinational clinical trial in relation to regional and national type 1 diabetes reference populations.

**Methods:**

Retrospective, cross-sectional evaluation of a subset of adolescent type 1 diabetes cardiorenal intervention trial (AdDIT) participants from Australia (*n* = 144), Canada (*n* = 312) and the UK (*n* = 173). Validated national measures of deprivation were used: the Index of Relative Socioeconomic Disadvantage (IRSD) 2016 (Australia), the Material Resources (MR) dimension of the Canadian Marginalisation index 2016 (Canada) and the Index of Multiple Deprivation (IMD) 2015 (UK). Representativeness was assessed by comparing the AdDIT cohort’s distribution of deprivation quintiles with that of the local paediatric type 1 diabetes population (regional), and the broader type 1 diabetes population for which the trial’s intervention was targeted (national).

**Results:**

Recruited study cohorts from each country had higher proportions of participants with higher SES, and significant underrepresentation of lower SES, in relation to their national references. The socioeconomic make-up in Australia mirrored that of the regional population (*p* = 0.99). For Canada, the 2nd least deprived (*p* = 0.001) and the most deprived quintiles (*p* < 0.001) were over- and under-represented relative to the regional reference, while the UK featured higher regional and national SES bias with over-representation and under-representation from the least-deprived and most-deprived quintiles (*p* < 0.0001).

**Conclusions:**

Significant national differences in trial participation of low SES participants were observed, highlighting limitations in access to clinical research and the importance of reporting sociodemographic representation in diabetes clinical trials.

**Trial registration:**

NCT01581476. Registered on 20 April 2012.

**Supplementary Information:**

The online version contains supplementary material available at 10.1186/s12916-023-03222-w.

## Background

Despite the increasing recognition that social determinants of health significantly impact clinical health outcomes, less than 15% of clinical trials published in leading medical journals report any data on participant socioeconomic status (SES) [1–3]. In the few studies reporting such data, participants of higher SES predominate with higher levels of reported household income and educational attainment [4–6]. The inclusion of participants into clinical trials from a wide variety of socioeconomic backgrounds is important to ensure that medical research innovations shown to be effective and safe have been evaluated and are thus generalizable to the broader clinic population [4]. This is particularly relevant in diabetes-related research, as many medication and medical device trials fail to report details relating to social or ethnic factors, or show low rates of participation of underrepresented groups [7].

Disadvantaged groups may experience barriers to high-quality care that include limited economic resources as well as limitations in accessing care centres due to obstacles in distance and time [8, 9]. In addition, many socially disadvantaged groups experience greater disease burden for whom clinical interventions may be more beneficial [10–12]. From a health equity perspective, individuals of lower SES should also be provided equal opportunity to participate in clinical research [13, 14].

There are few diabetes trials, particularly in paediatrics, reporting the participation of individuals with low SES [15]. The overall goal of this report was to examine the SES of participants recruited in the Adolescent type 1 Diabetes cardiorenal Intervention Trial (AdDIT). This study was conducted in three countries—Australia, Canada and the UK—with publicly funded healthcare systems as well as validated measures of SES to quantify social inequity within both the type 1 diabetes study participants and the general population. The specific study aims were to evaluate, within each individual country, how representative AdDIT study participants were in relation to (1) the local/regional population of persons with type 1 diabetes available for recruitment and (2) the national population of persons with type 1 diabetes.

## Methods

### Study population—AdDIT study cohort

AdDIT is an international (Canada, UK and Australia) study that evaluated adolescents for inclusion into an intervention (i.e. randomized control trial, RCT) or observational study arm between 2010 and 2016 [16]. Potential study participants for AdDIT were identified based on screening of albumin excretion (albumin-creatinine ratio, ACR) whereby high-risk subjects with increased albumin excretion (upper tertile of ACR) were eligible to participate in the intervention RCT, while those identified to be at low risk (lower and middle tertiles of ACR) were eligible to participate in the observational arm. ACR measurements were performed centrally at the WellChild Laboratory in London, England. Urine albumin was measured using nephelometric immunoassay according to the manufacturer’s instructions (Siemens BN Prospec). HbA1c was assessed locally using DCCT-aligned methods.

**Fig. 1:**
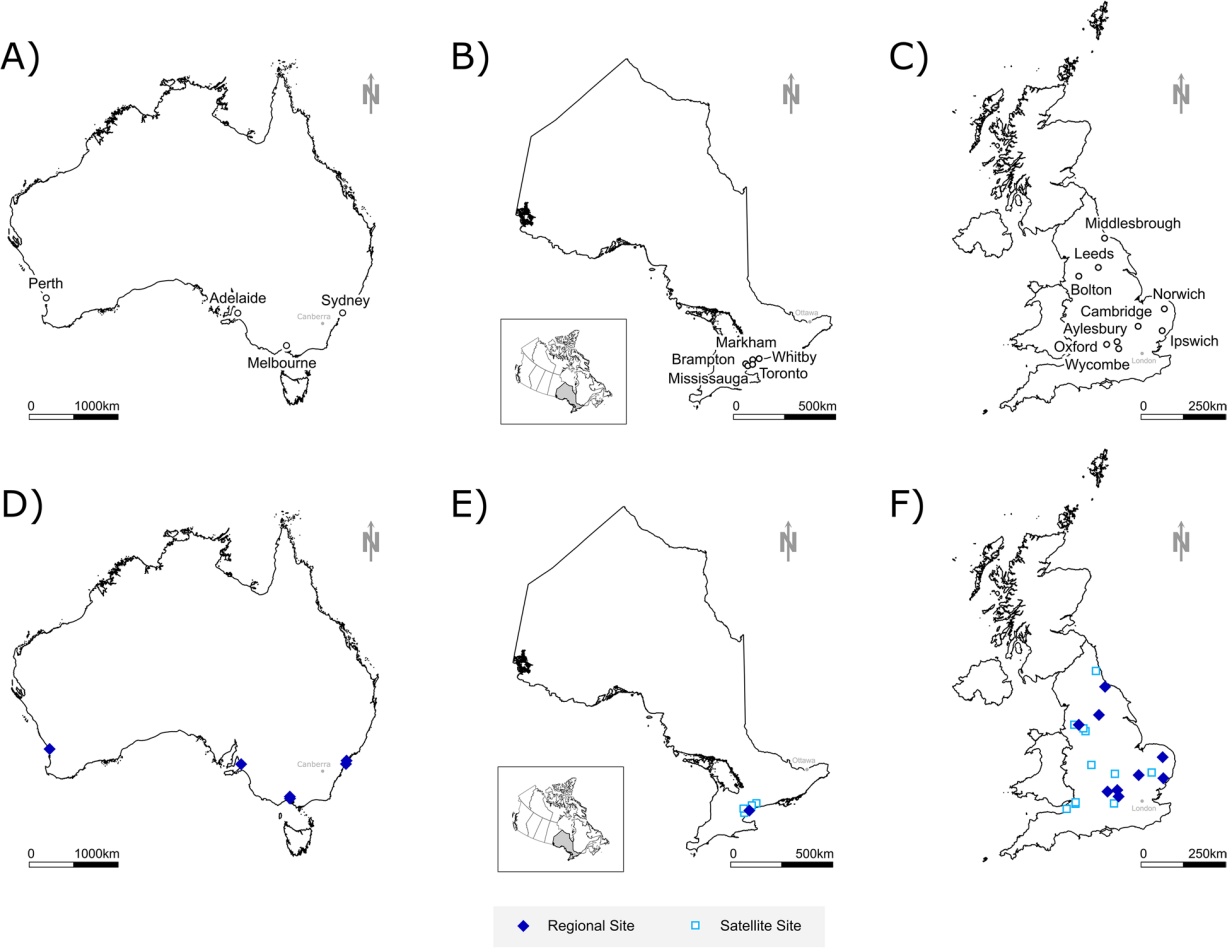
Distribution of AdDIT International sites across Australia, Canada and the UK. The top panels **A**–**C** present the urban centres in which AdDIT sites were located while the bottom panels **D**–**F** break down the type of study site. National capitals are presented in grey for reference

The current evaluation includes *N* = 631 of the 893 original AdDIT participants (70.4%) from both the intervention (*n* = 301/443; 67.9%) and observational (*n* = 330/450; 73.3%) study arms. Complete postal codes were available for participants from AdDIT Canada; however, local ethics restrictions pertaining to confidentiality limited access to postal code data for a subset of participants from AdDIT UK and AdDIT Australia, which did not allow the entire original AdDIT cohort to be evaluated as part of this study. Overall, the study included *n* = 144 of 201 from Australia, *n* = 312 of 323 from Canada and *n* = 175 of 369 from the UK (specifically England and Wales) (see Additional file 1: Fig. S1). Participant recruitment for AdDIT was completed at 6 sites across 4 Australian states for Australia, 5 sites in a single Canadian province (Ontario) for Canada and 23 sites across 8 of the 9 NHS health regions in England for the UK (see Fig. 1). This study was ethics approved at all study sites and the study protocol was in conformity with the Declaration of Helsinki.

### Deprivation indices

The study used validated, aggregate-level, area-based measures of deprivation and marginalization developed by the governments of each country to approximate individual-level SES. The indices from each country were analysed as quintile scores to allow comparisons with local/regional and national reference populations. For each country, measures of deprivation/marginalization were linked to participant data using postal codes as described by the guidance documents of each index. Postcodes of all *n* = 144 of the Australian participants were directly linked to the Socio-Economic Indices For Areas (SEIFA) 2016 including the Index of Relative Socio-economic Disadvantage (IRSD) [16]. For Canada, the 6-digit postal codes of all *n* = 312 participants were linked to dissemination area (DA) codes, the smallest census-based geographic unit, using Statistics Canada’s Postal Code Conversion File (PCCF) before being linked to the Material Resources (MR) dimension of the Canadian Marginalisation Index (CAN-Marg) 2016 [17]. For the UK, the postcodes for *n* = 173 of 175 participants were successfully linked to the Lower Layer Super Output Area (LSOA) code in which they were located. The LSOA code was subsequently linked to the Index of Multiple Deprivation (IMD) 2015 quintile score for *n* = 172 participants with an England-based postcode and the Welsh Index of Multiple Deprivation (WIMD) 2014 quintile score for *n* = 1 participant with a Wales-based postcode [18]. The postcodes of *n* = 2 participants from the UK could not be linked to their corresponding LSOA and were thus excluded from the present analysis. Both the IRSD and IMD measures were scored from Q1 (most disadvantaged/deprived) to Q5 (least disadvantaged/deprived), while the CAN-Marg quintiles were scored inversely with Q1 representing the least deprived quintile and Q5, the most deprived quintile.

### Local/regional and national paediatric type 1 diabetes reference populations

Aggregate data for both regional and national reference populations in England and Wales were extracted from the National Pediatric Diabetes Audit (NPDA) 2015–2016 and 2019–2020 Annual Report and Appendices [19]. The breakdown of paediatric T1D across IMD quintiles in the UK and in regions in which AdDIT sites were located were extracted directly from the NPDA reports.

Data from the paediatric type 1 diabetes population from the study’s main Canadian site, The Hospital for Sick Children (SickKids), and regional sites were used as the local/regional reference. Canadian reference data for paediatric type 1 diabetes were not readily available from government agencies at the national level as these data were consistently grouped alongside type 2 diabetes. In light of such a limitation, National references for paediatric type 1 diabetes in Canada were derived by combining data from the Pediatric Diabetes Network (PDN) and the Canadian Chronic Disease Surveillance System (CCDSS) [20, 21]. Due to the lack of linkage to CAN-Marg, the reference population for Canada was assumed to be evenly distributed across quintiles.

Regional reference data for 2015–2016 for Australian sites was obtained from the Australasian Diabetes Data Network (ADDN) in aggregate [22]. Postcodes from the ADDN dataset were linked to SEIFA 2016 indices using the method described above. Postcodes encompassing less than *n* = 4 participants were not included for confidentiality reasons. Nationally, aggregate prevalence data was extracted from the Australian Institute for Health and Wellness’s (AIHW) Diabetes 2020 Data Table 1.12, which broke down the prevalence of children and young adults aged 0 to 24 with type 1 diabetes in Australia (*n* = 20,664) across IRSD 2016 quintiles [22].

### Statistical analyses

All statistical analyses were completed using R Statistics v4.1.0 (www.r-project.org) Representativeness was first assessed overall by comparing the distribution of AdDIT participant cohorts across deprivation quintiles with that of their regional and national reference populations using chi-square tests. The distribution of the Australian cohort of AdDIT across IRSD quintiles was compared with the ADDN and AIHW references. The Canadian cohort of AdDIT was compared with the SickKids and inferred PDN/CCDSS references on the basis of their distribution across quintiles from the MR dimensions of the 2016 Can-Marg Index. The UK AdDIT cohort was compared with the regional and national NPDA references according to IMD 2015 quintiles. Confidence set at 95% (*α* = 0.05), such that *P*-values < 0.05 were deemed significant for this assessment.

Further testing assessing differences within quintiles was completed using Fisher’s Exact tests to evaluate over- and under-representation among quintile groups relative to their reference populations. To account for multiple testing within each deprivation index, a simple Bonferroni correction was used, whereby *P*-values < 0.005 (i.e. 0.05/10) were deemed significant. No comparisons were performed between the AdDIT cohorts from different countries; each cohort was analysed separately and compared with reference populations from their country using the country-specific indicators.

## Results

### AdDIT participant demographic data

The current study included *N* = 629 participants from the original AdDIT study for whom valid postal information was available. The assessed cohort was 46.9% female with a mean age of 14.1 ± 1.6 years, diabetes duration of 6.5 ± 3.2 years and mean HbA1c 8.5 ± 1.3% (69.2 ± 14.6 mmol/mol) (Table 1). Characteristics were clinically similar between countries aside from a higher proportion of insulin pump use (in relation to injection) in Canada, with 56.1% of Canadian participants reporting pump use as compared with Australia (36.8%) and the UK (35.1%); *P* < 0.001). Significant differences in self-identified ethnicity were seen, with a more diverse, non-White, ethnic composition seen in Canada (40.9%) in relation to the UK (6.2%) or Australia (4.7%) (*P* < 0.001). The proportion of participants from the interventional and observational arms of AdDIT did not differ across deprivation quintiles and were analysed as a single cohort as part of this study (see Additional file 1: Fig. S2).

**Table 1:** Baseline characteristics of AdDIT participants presented overall and by country

Baseline characteristics	Units	Overall	AdDIT International			*P*
			**Australia**	**Canada**	**UK**	
*N*	-	629	144	312	173	< 0.0001
**Demographics**						
Age	Years	14.1 ± 1.6	13.8 ± 1.5	14.3 ± 1.6	13.8 ± 1.6	0.004
Sex	% Female	46.8%	54.9%	47.1%	39.5%	0.025
	% Male	53.2%	45.1%	52.9%	60.5%	
Diabetes duration	years	6.4 ± 3.2	6.1 ± 2.7	6.6 ± 3.4	6.5 ± 3.3	0.265
Ethnicity	% White	78.6%	93.8%	59.3%	95.4%	< 0.0001
	% Black	2.8%	0.0%	6.7%	0.6%	
	% South Asian	4.7%	1.4%	8.3%	1.2%	
	% Chinese	3.0%	0.0%	6.7%	0.0%	
	% Aboriginal	0.4%	0.7%	0.3%	0.0%	
	% Other	10.6%	4.2%	18.6%	2.9%	
**Diabetes management**						
Insulin regimen	% MDI	45.9%	63.2%	43.9%	64.9%	< 0.0001
	% CSII	54.1%	36.8%	56.1%	35.1%	
HbA1c	%	8.5 ± 1.3	8.6 ± 1.3	8.4 ± 1.3	8.4 ± 1.4	0.459
	mmol/mol	69.2 ± 14.5	70.4 ± 14.6	69.2 ± 14.0	68.2 ± 15.7	
ACR	mmol/L	1.54 ± 1.14	2.07 ± 1.25	1.53 ± 1.21	1.11 ± 0.61	< 0.0001

Continuous variables are presented as mean ± SD, while categorical variables are presented as percentages (%)

### Comparisons with regional paediatric type 1 diabetes references

**Fig. 2:**
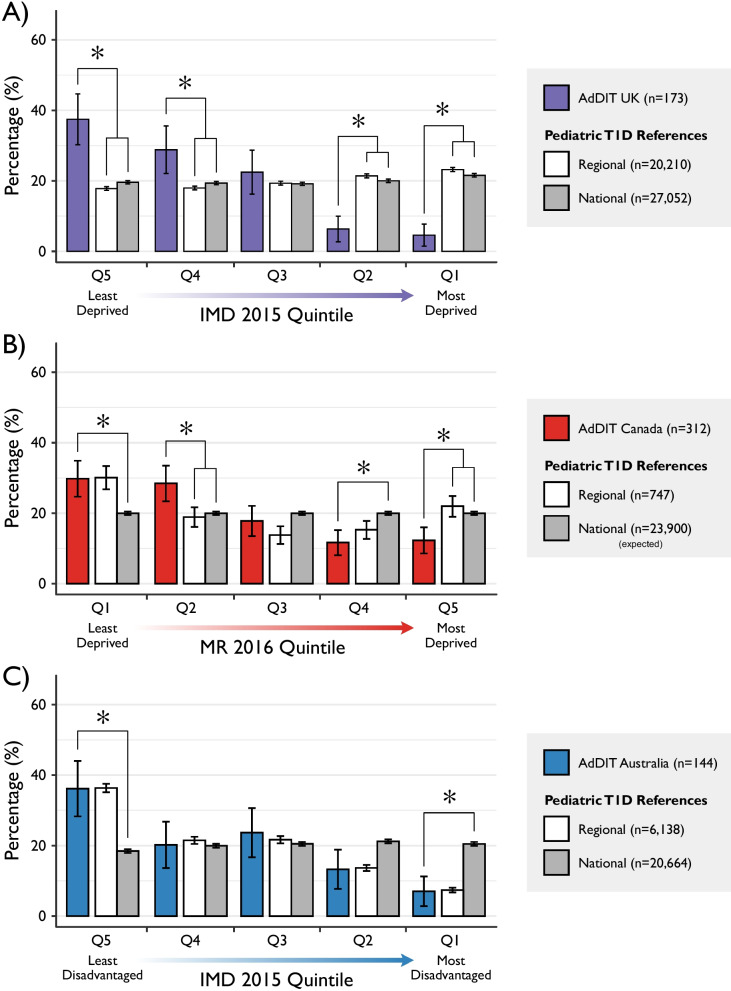
Distribution of the UK, Canadian and Australian cohorts of AdDIT. **A** Distribution of the UK-based cohort of AdDIT, the NPDA regional reference and the NPDA national reference across quintiles of the Index of Multiple Deprivation (IMD) 2015. **B** Distribution of the Canadian cohort of AdDIT, the SickKids regional reference and the derived national reference across quintiles of the Material Resources (MR) 2016. **C** Index of Relative Socioeconomic Disadvantage (IRSD) 2016. Differences between the AdDIT cohort and both reference populations are presented. Error bars represent 95% confidence intervals

The distribution of participants from the UK across IMD quintiles differed considerably when compared with the regional NPDA 2015–2016 reference population (*P* < 0.0001), as depicted in Fig. 2. Substantial variation in SES was observed among the AdDIT UK cohort, with a strong skew toward higher SES. Participants from both the least (37.6% vs. 17.9%; *P* < 0.0001) and 2nd least (28.9% vs. 18.0%; *P* < 0.001) deprived quintiles were greatly overrepresented when compared with the regional NPDA reference. Inversely, participants from the most (4.6% vs. 23.3%; *P* < 0.0001) and 2nd most (6.4% vs. 21.5%; *P* < 0.0001) deprived quintiles were underrepresented relative to the NPDA references.

The Canadian AdDIT cohort was found to be significantly different than the local paediatric type 1 diabetes population reference with respect to their distribution across quintiles of the MR dimension (*P* < 0.0001). When examined further at the quintile level, the difference observed based on MR was an over-representation and under-representation of participants from the 2nd least deprived quintile (28.2% vs. 18.9%; *P* = 0.001) and under-representation of participants from the most deprived quintile (12.2% vs. 22.0%; *P* < 0.001) relative to their regional reference, respectively. While it did not reach significance, a slight overrepresentation of participants from the Q3 was observed relative to the regional reference (18.6% vs. 13.8%; *P* = 0.049).

The overall distribution of the Australian AdDIT cohort across IRSD (*P* = 0.98) quintiles was not significantly different when compared with the ADDN paediatric type 1 diabetes reference population. No additional within-quintile differences between the AdDIT Australia cohort and the ADDN reference were observed.

### Comparisons with national paediatric type 1 diabetes references

The UK AdDIT cohort was also found to differ significantly relative to the national NPDA reference population with respect to their distribution across IMD 2015 quintiles (*P* < 0.0001). Similar to their regional findings, participants from both the least (37.6% vs. 19.7%; *P* < 0.0001) and 2nd least (28.9% vs. 19.4%; *P* = 0.0027) deprived quintile groups were considerably overrepresented, and the most (4.6% vs. 21.6%; *P* < 0.0001) and 2nd most (6.4% vs. 20.1%; *P* < 0.0001) deprived quintiles were underrepresented, relative to the national NPDA reference.

The overall distribution of the Canadian AdDIT cohort was also skewed relative to their derived national reference. For the MR dimension, a greater proportion of individuals from less marginalized areas, and a lower proportion of individuals from areas characterized by high marginalization, was observed. Individuals from the least (29.5%; *P* < 0.0001) and 2nd least (28.2%; *P* < 0.0001) marginalized quintile of the MR dimension were significantly overrepresented. Likewise, underrepresentation of individuals from the most (12.2%; *P* < 0.001) and 2nd most (11.5%; *P* < 0.001) marginalized quintiles was observed for the MR dimension.

Significant differences were observed between the Australian AdDIT cohort and AIHW reference population with respect to the IRSD 2016 index (*P* < 0.0001). Relative to their national reference population from the AIHW, on the basis of IRSD 2016 quintiles, the Australian AdDIT cohort was found to have a significantly greater proportion of individuals from the least deprived (36.1% vs. 18.3%; *P* < 0.0001) quintile group and a significantly lower proportion of individuals from the most (6.9% vs. 20.4%; P < 0.0001) deprived quintile. The 2nd most deprived quintile was also slightly underrepresented in the AdDIT Australia cohort (13.2%) compared the to national AIHW reference (21.1%), although this difference did not reach the threshold for significance (*P* = 0.018).

## Discussion

In this international paediatric type 1 diabetes study, we report significant differences in SES between recruited study participants and reference regional and national populations with type 1 diabetes, within each of the AdDIT countries (see Table 2). Regional differences were seen between the three countries evaluated, such that research participation in the UK and Canada was overall under-represented for more deprived SES quintiles, while research participation in Australia matched their regional type 1 diabetes population. On a macro level, comparisons with all national reference populations showed that the recruited AdDIT study cohort was not representative of the overall population with type 1 diabetes.

**Table 2:** Summary of recruitment from AdDIT countries

	**AdDIT UK**	**AdDIT Canada**	**AdDIT Australia**
**# of sites**	20 sites	5 sites	7 sites
**Regions/provinces/states**	8	1	4
**Regional representation pattern**	Not representative	Partially representative	Fully representative
	Over-recruitment of high SES and under-recruitment of low SES	Under-recruitment of low SES	Recruitment consistent with regional population
**Recruitment approach**	Larger number of diabetes centres from wider geographic area with fewer participants recruited per clinic	Diabetes centres from a single urban area	Diabetes centres from 4 large urban areas
**Interpretation**	Potential for selection bias in participants with greater interest, time or perceived suitability (Higher SES)	Selection bias in background urban population with higher proportions of high and low SES with a “U shaped” distribution consistent with urban income polarization	Skew in regional population consistent with census data showing that most advantaged population quintiles are clustered around Australian cities and selected coastal areas
**National representation**	Not representative	Not representative	Not representative

Different recruitment approaches were used in each country. In the UK, a larger number of recruitment sites including towns and urban metropolitan areas were active from a wider geographic area. While this approach was intended to include a broader enrollment base, it meant that fewer participants per center were recruited (see Fig. 1). This may have contributed to resulted in a selection bias towards higher SES participants, who may have volunteered or have been preferentially selected based on greater interest, time or ability and perceived suitability for clinical research. Canada and Australia recruited from fewer sites, primarily represented by larger clinics in more urban areas. Canadian recruitment was centred in the Greater Toronto metropolitan area, and while recruitment was representative at higher SES quintiles, there was an underrepresentation of lower SES. Interestingly, the regional reference type 1 diabetes population in Canada shows a classic U-shaped curve of income polarization, whereby there is a loss of the middle SES quintiles and an over-representation of high and low marginalization. Income inequality is quantified using the Gini index, and Toronto has the highest national urban Gini index for Canada (https://www150.statcan.gc.ca/n1/daily-quotidien/220713/g-d007-eng.htm.). This trend of income polarization has intensified in Canadian and other cities globally alongside a progressive loss of middle-income earners in urban areas [23, 24]. Australian regional data were collected in moderate-sized clinics in many regional cities and describe a population weighted towards a higher SES overall, with much lower proportions of participants with greater deprivation/lower SES than Canada or the UK. Recruitment was highly representative of the regional population but reflects that, in Australia, the most advantaged SES quintiles are clustered around capital cities and selected coastal areas, while the most disadvantaged quintiles are in more remote and regional areas [25, 26].

Strengths of this report are that validated SES measures from publicly funded health care systems were used, which allowed for descriptive international comparisons that lessen issues related to participant health insurance status. In addition, this allows for a nuanced evaluation of SES and study enrolment, allowing for comparisons with both regional and national populations of individuals with type 1 diabetes. In addition, while these data report SES differences in study recruitment, they also show the engagement of participants across a wide range of socioeconomic backgrounds. Strategies to optimize convenience and the overall participant experience at sites included flexibility in data collection by combining study and diabetes clinic visits, the availability of early or late or weekend appointments, reimbursement for travel and the availability of shared health care for participants living away from some sites [6, 27]. In the AdDIT study, weekend and after-hours visits and shared care strategies were implemented at some sites based upon advice provided by participant and parent/caregiver advisory group who emphasized this need, in particular, for two-household families and parents with restrictive employment schedules. AdDIT was also conducted prior to the more recent wider adoption of virtual research visits, which may allow for greater engagement of the underserved [8, 28–30].

This study has some limitations. Only a subset of the original AdDIT cohort could be evaluated in the current study due to local ethics restrictions at certain sites/countries. This could have potentially impacted our results, particularly for AdDIT UK, as a large portion of the cohort could not be assessed. We used population-level measures developed by national agencies to quantify social inequity within populations that are created by agglomerating multiple socioeconomic and environmental census data sources. It is important to recognize that individual national indexes vary with regard to specific components used to assess marginalization and SES and this is the reason why data in this report are reported and described separately for each country [17]. While area-based measures of SES may not be an ideal proxy for individual-based SES measures, they are valuable in describing socioeconomic trends where participant-level data are not available [31, 32]. Our study was limited to aggregate-level data for most regional and national reference groups, limiting our ability to evaluate and compare clinical characteristics such as HbA1c, ACR and insulin therapy. This report also has limited data on the impact of ethnicity on recruitment, as this data was not universally available from regional and national sources. Lastly, our study applied an assumption of uniform SES distribution for paediatric type 1 diabetes in Canada due to limited reference data at the national level, which may not reflect the true distribution of SES for this population.

## Conclusions

As a large, multicenter trial, this report is unique as it describes socioeconomic details of clinical trial participants and how they compare with the populations from which they were sampled. This report also highlights the realities and challenges of contemporary clinical research in the context of broader social and economic inequities. Around 40–60% of clinical trials have insufficient or delayed recruitment, and investigators are often challenged to counterpoise the pressures of recruitment using more available, higher SES participants with those who are more representative [33–35]. Elements to consider in trial design would include snowball or social network-based approaches and community partnerships in addition to flexible data collection strategies [6, 33]. Trial recruitment and conduct should include personalized, needs-based approaches that include appropriate financial compensation and address cultural and language barriers to participation. These strategies are important to not only increase the level of involvement and participation in health research for disadvantaged groups, but also ensure the outcomes and implementation of health research are relevant.

### Supplementary Information

**Additional file 1:** **Table S1.** Country-specific measures of deprivation, and sources of regional and national reference type 1 diabetes population data. **Table S2.** Raw counts and proportions of the UK, Canadian and Australian cohorts of AdDIT across deprivation quintiles. Intra-quintile differences are presented. **Figure S1.** Flowchart of study participants from the original AdDIT UK, AdDIT Canada and AdDIT Australia cohorts included in the current study. **Figure S2.** Distribution of the Experimental and Observational study arms among the A) AdDIT UK and B) AdDIT Canada cohorts across country-specific deprivation quintiles.

## Data Availability

The datasets generated and/or analysed during the current study are not publicly available but reasonable requests may be forwarded to the AdDIT Steering Committee for review.

## Abbreviations

ACR: Albumin-creatinine ratio
AdDIT: Adolescent type 1 Diabetes cardiorenal Intervention Trial
ADDN: Australasian Diabetes Data Network
AIHW: Australian Institute for Health and Wellbeing
CCDSS: Canadian Chronic Disease Surveillance System
CAN-Marg: Canadian Marginalisation Index
IMD: Index of Multiple Deprivation
IRSD: Index of Relative Socioeconomic Disadvantage
LSOA: Lower Layer Super Output Area
MR: Material Resources
NHS: National Health Service
NPDA: National Pediatric Diabetes Audit
PCCF: Postal Code Conversion File
PDN: Pediatric Diabetes Network
SEIFA: Socio-Economic Index For Areas
SES: Socioeconomic status
UK: United Kingdom
WIMD: Welsh Index of Multiple Deprivation
