# Supplementary material for: Socioeconomic representativeness of Australian, Canadian and British cohorts from the paediatric diabetes AdDIT study: comparisons to regional and national data
Source: BMC Med. 2023 Dec 20;21:506. doi: 10.1186/s12916-023-03222-w (PMC10734126; doi:10.1186/s12916-023-03222-w)

ADDITIONAL FILE 1

Table S1. Country-specific measures of deprivation, and sources of regional and national reference type 1 diabetes population data.

Characteristics	AdDIT UK	AdDIT Canada	AdDIT Australia
**Deprivation Measure**	Index of Multiple Deprivation (IMD) 2015	Canadian Marginalization Index (CAN-Marg) 2016	Socioeconomic Indices for Areas (SEIFA) 2016
**Sub-Domain**	N/A	Material Resources (MR)	Index of Relative Socioeconomic Disadvantage (IRSD)
**Geographic Unit**	Lower-layer Sorting Output Area (LSOA)	Dissemination Area (DA)	Postcode Area (POA)
**Pediatric Type 1 Diabetes References**	UK	Canada	Australia
**Regional**	National Pediatric Diabetes Audit (NPDA) 2015-16 & 2019-20	Regional data from the Hospital for Sick Children	Australasian Diabetes Data Network (ADDN)
**National**	National Pediatric Diabetes Audit (NPDA) 2015-16	Canadian Chronic Disease Surveillance System (CCDSS) 2016 & Pediatric Diabetes Network 2017-18	Australian Institute of Health & Welfare Data (AIHW) 2018

Table S2: Raw counts and proportions of the UK, Canadian and Australian cohorts of AdDIT across deprivation quintiles. Intra-quintile differences are presented.

AdDIT Cohort	Reference Population	Type	Index	Quintile	AdDIT		Reference		OR	95% CI		P
					N	%	N	%		Low	High	
UK	NPDA	National	IMD 2015	Q1 (most)	8	*4.6*	5,823	*21.6*	0.176	0.075	0.354	<0.0001
				Q2	11	*6.4*	5,428	*20.1*	0.271	0.132	0.497	<0.0001
				Q3	39	*22.5*	5,198	*19.2*	1.224	0.833	1.762	0.247
				Q4	50	*28.9*	5,256	*19.4*	1.686	1.187	2.364	0.0027
				Q5 (least)	65	*37.6*	5,317	*19.7*	2.460	1.777	3.383	<0.0001
				Total	173	-	27,052	-				
	NPDA subset	Regional	IMD 2015	Q1 (most)	8	*4.6*	4,700	*23.3*	0.160	0.068	0.323	<0.0001
				Q2	11	*6.4*	4,338	*21.5*	0.248	0.122	0.457	<0.0001
				Q3	39	*22.5*	3,921	*19.4*	1.209	0.822	1.742	0.287
				Q4	50	*28.9*	3,641	*18.0*	1.850	1.301	2.595	<0.001
				Q5 (least)	65	*37.6*	3,609	*17.9*	2.768	1.999	3.808	<0.0001
				Total	173	-	20,210	-				
Canada	PDN/CCDSS	National	MR 2016	Q1 (least)	92	*29.5*	4,780	*20.0*	1.673	1.294	2.148	<0.0001
				Q2	88	*28.2*	4,780	*20.0*	1.571	1.211	2.024	<0.001
				Q3	58	*18.6*	4,780	*20.0*	0.913	0.673	1.221	0.569
				Q4	36	*11.5*	4,780	*20.0*	0.522	0.357	0.741	<0.001
				Q5 (most)	38	*12.2*	4,780	*20.0*	0.555	0.384	0.782	<0.001
				Total	312	-	23,900	-				
	SickKids	Regional	MR 2016	Q1 (least)	92	*29.5*	225	*30.1*	0.970	0.717	1.307	0.883
				Q2	88	*28.2*	141	*18.9*	1.688	1.225	2.319	0.001
				Q3	58	*18.6*	103	*13.8*	1.427	0.984	2.057	0.049
				Q4	36	*11.5*	114	*15.3*	0.724	0.471	1.094	0.122
				Q5 (most)	38	*12.2*	164	*22.0*	0.493	0.328	0.729	<0.001
				Total	312	-	747	-				
Australia	AIHW	National	IRSD 2016	Q1 (most)	10	*6.9*	4,207	*20.4*	0.292	0.137	0.554	<0.0001
				Q2	19	*13.2*	4,361	*21.1*	0.568	0.331	0.926	0.0182
				Q3	34	*23.6*	4,209	*20.4*	1.209	0.796	1.793	0.350
				Q4	29	*20.1*	4,099	*19.8*	1.019	0.652	1.545	0.917
				Q5 (least)	52	*36.1*	3,788	*18.3*	2.518	1.753	3.583	<0.0001
					144	-	20,664					
	ADDN	Regional	IRSD 2016	Q1 (most)	10	*6.9*	445	*7.2*	0.955	0.444	1.828	0.999
				Q2	19	*13.2*	831	*13.5*	0.971	0.562	1.591	0.999
				Q3	34	*23.6*	1,324	*21.6*	1.123	0.738	1.673	0.540
				Q4	29	*20.1*	1,312	*21.4*	0.928	0.592	1.411	0.837
				Q5 (least)	52	*36.1*	2,226	*36.3*	0.993	0.690	1.417	0.999
				Total	144	-	6,138	-				

Figure S1. Flowchart of study participants from the original AdDIT UK, AdDIT Canada and AdDIT Australia cohorts included in the current study.

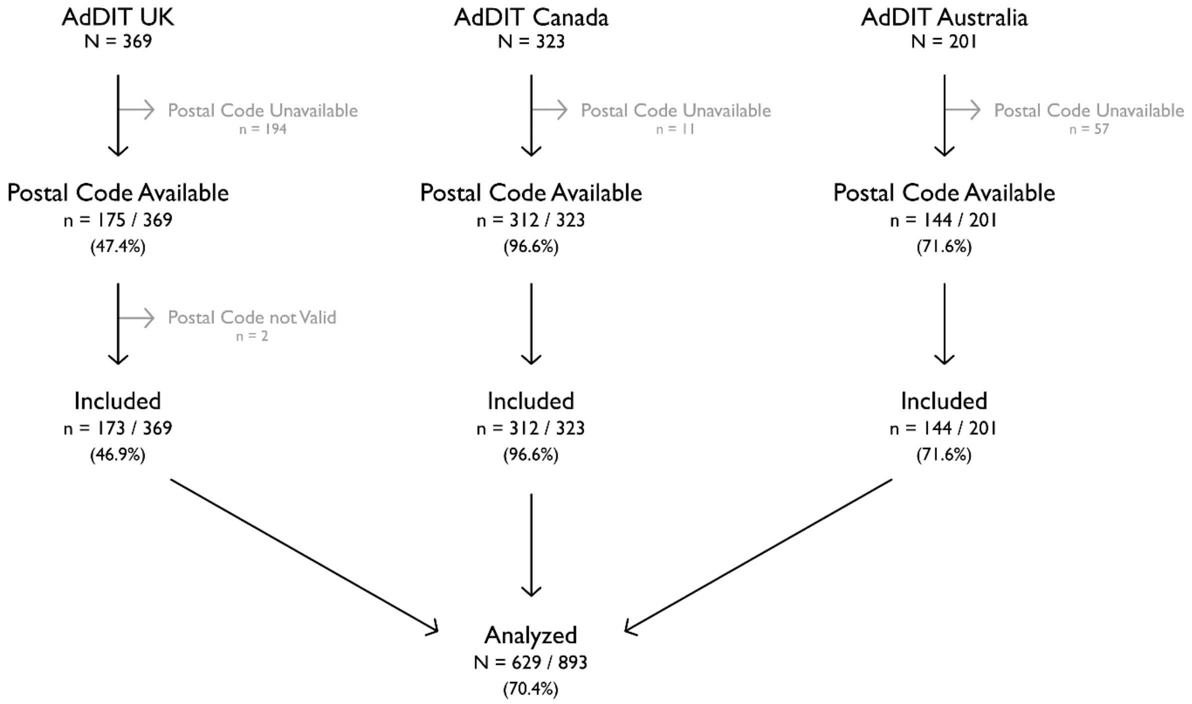

Figure S2. Distribution of the Experimental and Observational study arms among the A) AdDIT UK and B) AdDIT Canada cohorts across country-specific deprivation quintiles. Bars in colour represent the Experimental Arm whereas bars in white represent the Observational Arm. All participants of the AdDIT Australia cohort were part of the Experimental Arm and thus not included.

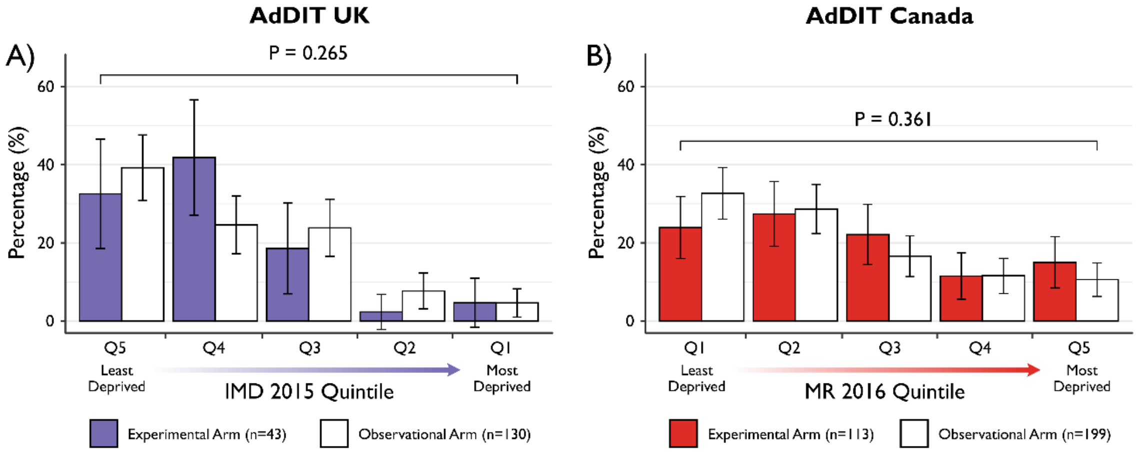

Supplement: Supplementary file 1 — Additional file 1: Table S1. Country-specific measures of deprivation, and sources of regional and national reference type 1 diabetes population data. Table S2. Raw counts and proportions of the UK, Canadian and Australian cohorts of AdDIT across deprivation quintiles. Intra-quintile differences are presented. Figure S1. Flowchart of study participants from the original AdDIT UK, AdDIT Canada and AdDIT Australia cohorts included in the current study. Figure S2. Distribution of the Experimental and Observational study arms among the A) AdDIT UK and B) AdDIT Canada cohorts across country-specific deprivation quintiles. [file 12916_2023_3222_MOESM1_ESM.pdf]
